# The Difference in Prognosis between Renal Sinus Fat and Perinephric Fat Invasion for pT3a Renal Cell Carcinoma: A Meta-Analysis

**DOI:** 10.1371/journal.pone.0149420

**Published:** 2016-02-18

**Authors:** Zhiling Zhang, Chunping Yu, Liliya Velet, Yonghong Li, Lijuan Jiang, Fangjian Zhou

**Affiliations:** 1 Department of Urology Sun Yat-Sen University Cancer Center, Guangzhou, China; 2 State Key Laboratory of Oncology in South China, Guangzhou, China; 3 Collaborative Innovation Center for Cancer Medicine, Guangzhou, China; 4 Department of Nephrology, Guangdong General Hospital, Guangdong Academy of Medical Sciences, Guangzhou, China; 5 Northeast Ohio Medical University, Rootstown, Ohio, United States of America; Shanghai Jiao Tong University School of Medicine, CHINA

## Abstract

**Background:**

In the current Tumour-Node-Metastasis (TNM) classification system for renal cell carcinoma (RCC), both renal sinus fat invasion (SFI) and perinephric fat invasion (PFI) are defined as T3a, suggesting that the prognosis should be similar for the two pathologic findings. Several studies, however, have reported a worse prognosis for SFI in patients with a T3a tumor. In order to compare the prognosis of these two pathologic findings (SFI versus. PFI) in a more comprehensive way, this meta-analysis was performed.

**Methods:**

To identify relevant studies, Medline, Embase, Cochrane Library, and Scopus database were searched from the inception until October 2014. A meta-analysis was performed using Review Manager 5.2 and STATA 11. Pooled Odds ratio (OR) and/or hazard ratio (HR) with 95% confidence interval (CI) were calculated to examine the risk or hazard association.

**Results:**

A total of 6 studies including 1031 patients qualified for analysis. T3a RCC patients with SFI were significantly associated with poor cancer specific survival(CSS) (HR: 1.47, 95% CI: 1.19–1.83; P<0.001) compared to those with PFI. In T3aNx/N0M0 subgroup, SFI patients also showed a worse prognosis than those with PFI (CSS, HR: 1.94, 95% CI: 1.21–3.12; P = 0.006). T3a RCC patients with SFI had higher Furhman grade, greater possibility of lymph node metastasis, sarcomatoid differentiation and tumour necrosis. Main limitation is the relatively small number of included studies.

**Conclusion:**

The present meta-analysis suggested that SFI is associated with worse CSS in patients with pT3a RCC. However, due to the small number of included studies, future studies with a large sample size are required to further verify our findings.

## Introduction

In the 2002 Tumour-Node-Metastasis (TNM) classification system, T3 renal cell carcinoma (RCC) included tumors that extend to fat, adrenal and major veins [1]. The 2009 TNM classification system made changes to the T3 stage in order to make it more powerful in differentiating prognosis [2]. For example, renal vein invasion was down-staged from T3b to T3a and adrenal invasion was up-staged from T3a to T4 or M1. Even so, there are still controversies on the current TNM classification system, especially the T3a stage.

There are two kinds of pathological findings in the current T3a fat invasion RCC: renal sinus fat invasion (SFI) and perinephric fat invasion (PFI). From an anatomical perspective, RCC with SFI is more likely to develop metastasis and may be associated with worse prognosis [3]. Firstly, renal sinus has no capsule, which serves as a barrier to extra-renal spread. Secondly, abundant veins and lymphatics in the renal sinus provide increased opportunity for tumor dissemination. Conversely, in perinephric fat the density of micrangium and lymphatics is much lower than in the sinus fat. Hence, theoretically, the risk of metastasis should be lower and the prognosis better in PFI patients than those with SFI. After careful literature review, we found that this topic is still under controversy [4–8]. Some studies report that patients with T3a RCC with PFI have significant survival superiority than those with SFI, while others failed to find a significant difference in survival between the two groups. Due to the rarity of T3a RCC, the significance of SFI has not been clearly established. Meanwhile, the statistical power of the individual studies is rather limited. Thus, a pooled analysis with a large sample size is needed to better illuminate this issue. In the present study, we conducted a meta-analysis of all the published literature about SFI and PFI in T3a fat invasion RCC. In so doing, we aimed to comprehensively compare the difference in prognosis between SFI and PFI.

## Methods

### Literature search strategy

We conducted a search of the Medline, Embase, Cochrane Library and Scopus databases to identify studies until Oct 24, 2014. Search terms included terms for kidney cancer ("renal cancer", "kidney cancer", "renal cell carcinoma", "renal neoplasm", "renal malignancy"), sinus fat ("sinus fat", "hilum fat", "hilus fat) and perinephric fat ("perinephric fat", "perirenal fat"). Furthermore, references of retrieved articles and reviews were manually screened for additional studies.

### Inclusion and exclusion criteria

The following criteria for eligibility were set before collecting articles: (1) articles were published in English. (2) studies focused on T3a RCC without adrenal gland and renal vein invasion. (3) the prognosis was compared between SFI and PFI in T3a RCC patients. The following studies were excluded from the analyses: (1) letters, reviews and conference abstracts. (2) studies in which necessary data were not provided to estimate hazard ratio (HR) and 95% confidence interval (CI). Patients with SFI plus PFI were considered as being within the SFI group.

### Data extraction and quality assessment

Two investigators (Zhang ZL and Yu CP) independently reviewed the eligible studies and extracted the data. Disagreements were resolved by discussion among all authors. The quality of the included studies was assessed by the Newcastle-Ottawa Scale (NOS)[9]. Studies with seven or more stars were defined as high quality studies.

### Statistical analysis

The impact of SFI compared with PFI on cancer specific survival(CSS) was measured by the combined HRs and their 95% CIs extracted from each eligible study. If these statistical variables were not available in an article, we estimated them from the given data utilizing methods described by Tierney and colleagues [10]. By convention, an observed HR>1 implied worse survival for SFI patients. Odds ratio (OR) was used to evaluate the association between SFI and clinicopathologic features. All numbers needed for calculating OR and their 95% CIs were directly extracted from data provided in the included studies. The heterogeneity among the studies was assessed by a chi-square-based Q statistic test, and the I^2^ value was used to quantify the heterogeneity (I^2^ = 0–50%, no or moderate heterogeneity; I^2^>50%, significant heterogeneity). Fixed-effect model was used if there was no significant heterogeneity (P<0.10 for the Q test). Otherwise, the random-effect model was used [11]. Egger’s test and Begg’s test were performed to identify the possibility of publication bias [12–13]. The robustness of the pooled results was confirmed by one-removed analysis in which the data of an individual study were removed each time. All P values were two-tailed and the P value<0.05 was considered statistically significant. The statistical analysis was conducted using the Review Manager 5.2 (Cochrane Collaboration, Oxford, UK) and STATA 11(StataCorp, College Station, Texas, USA).

## Results

### Study characteristics

**Fig 1:**
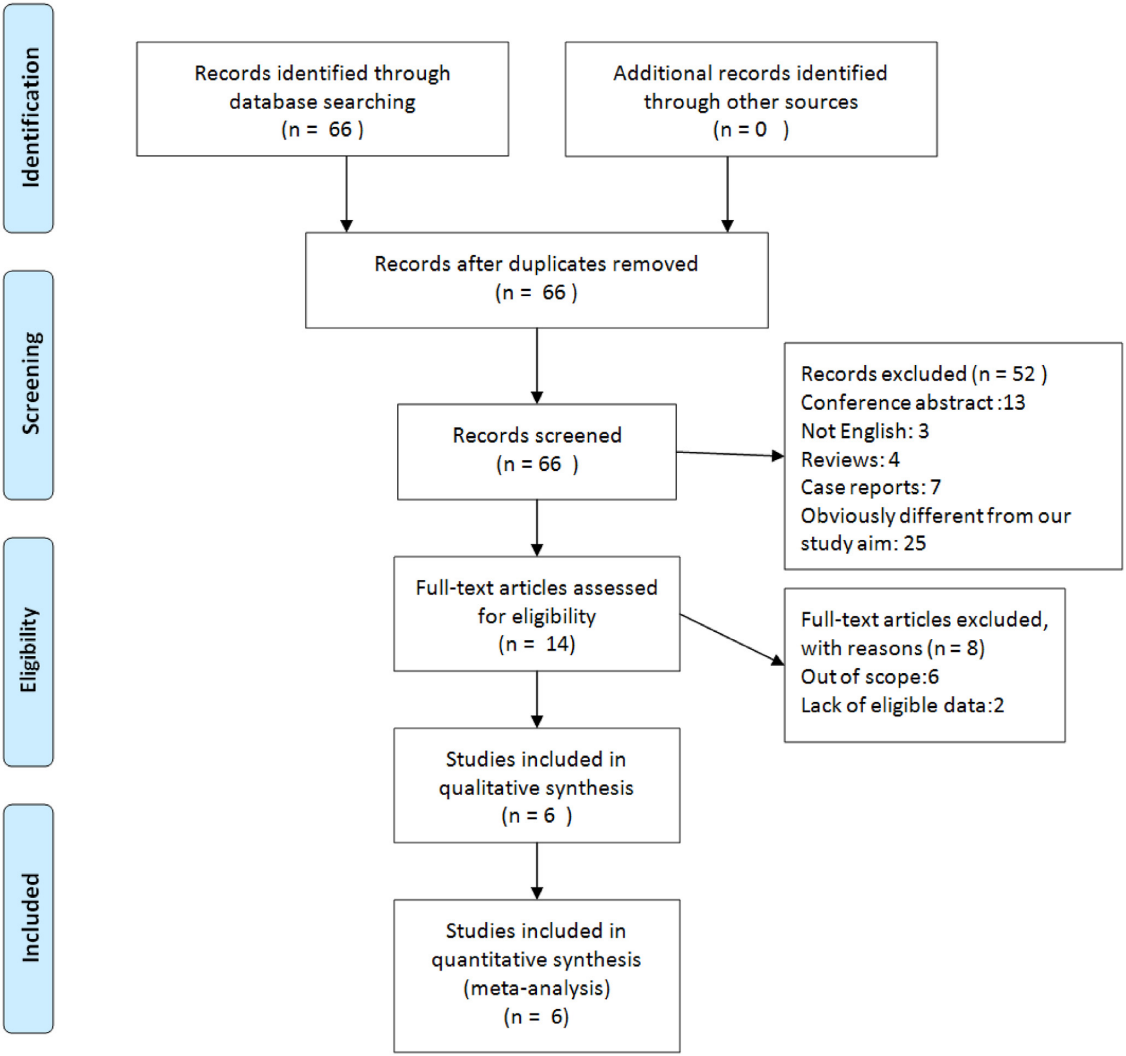
Flow diagram of studies selection procedure.

A total of 66 studies were initially identified from the above mentioned four databases by using the given search terms. No duplicated publications were found, so the 66 studies were screened by reading the title and abstract, after which 52 studies were excluded (conference abstract: 13; not English: 3; reviews: 4; case reports: 7; obviously different from our study aim: 25). Full texts of the remaining 14 studies were inspected for additional screening. Eight studies were excluded because 6 were out of scope and 2 lacked of eligible data. Finally, 6 articles met the inclusion criteria and were included in this meta-analysis (Fig 1).

We summarized the major characteristics of the 6 studies in Table 1. All six eligible articles compared the prognostic difference between SFI and PFI in pT3a RCC. One of the 6 included studies only focused on T3aNx/N0M0 [14]; one only reported HR for overall T3a patients [5]; the remaining four studies reported data on both overall T3a patients and T3aNx/N0M0 subgroup [4, 6–8], however in one of them, in the T3aNx/N0M0 subgroup, the HR could not be calculate [7]. All six studies utilized CSS to assess the prognostic difference between SFI and PFI. Only one study used disease free survival in T3aN0x/N0M0 subgroup as an indicator [4]. HRs and 95% CIs were directly extracted from five studies and calculated from survival curves in one study [4–8, 14]. The meta-analysis on the relationship between SFI and clinicopathologic parameters was performed based on 5 studies, which had eligible data for this analysis [4–8].

**Table 1:** Characteristics of the Included Studies.

First author	Year	Country	Recruitment period	Study design	Age (median)	Follow-up (mean)	Surgery type	No. all RCC pts	No. T3a Pts (%)	No.PFI	No.SFI	Outcomes measured (CSS)	NOS score
Portela^[^^14^^]^	2011	Spain	2000–2004	retrospective	60.0	30.6m	NA	260	20(7.7)	11	9	T3aNx/N0M0: Curve estimated HR	7
Bertini^[^^4^^]^	2009	Italy	1989–2006	retrospective	63.0	38.0m	RN	1282	105(8.2)	70	35	Overall T3a and T3aNx/N0M0: Multivariable HR reported	8
Poon^[^^8^^]^	2008	USA	1988–2007	retrospective	64.5	24.0m	RN+PN	1244	230(18.5)	167	63	Overall T3a and T3aNx/N0M0: Multivariable HR reported	9
Bedke^[^^6^^]^	2008	Germany	1990–2007	retrospective	61.8	34.8m	RN+PN	1183	106(9.0)	58	48	Overall T3a: Univariable HR reported T3aNx/N0M0: Multivariable HR reported	7
Margulis^[^^7^^]^	2007	USA	1990–2006	retrospective	58.2	33.5 m	RN+PN	3470	365(10.5)	199	166	Overall T3a: Univariable HR reported	7
Thompson^[^^5^^]^	2005	USA	1970–2002	retrospective	NA	72.0m	RN+PN	>4000	205(5.1)	162	43	Overall T3a: Multivariable HR reported	9

NA: not available; RN: radical nephrectomy; PN: partial nephrectomy; RCC: renal cell carcinoma; pts: patients; PFI: perinephric fat invasion; SFI: renal sinus fat invasion; CSS: cancer specific survival; HR: hazard ratio; NOS, Newcastle-Ottawa Scale.

### SFI and CSS in T3a RCC patients with fat invasion

**Fig 2:**
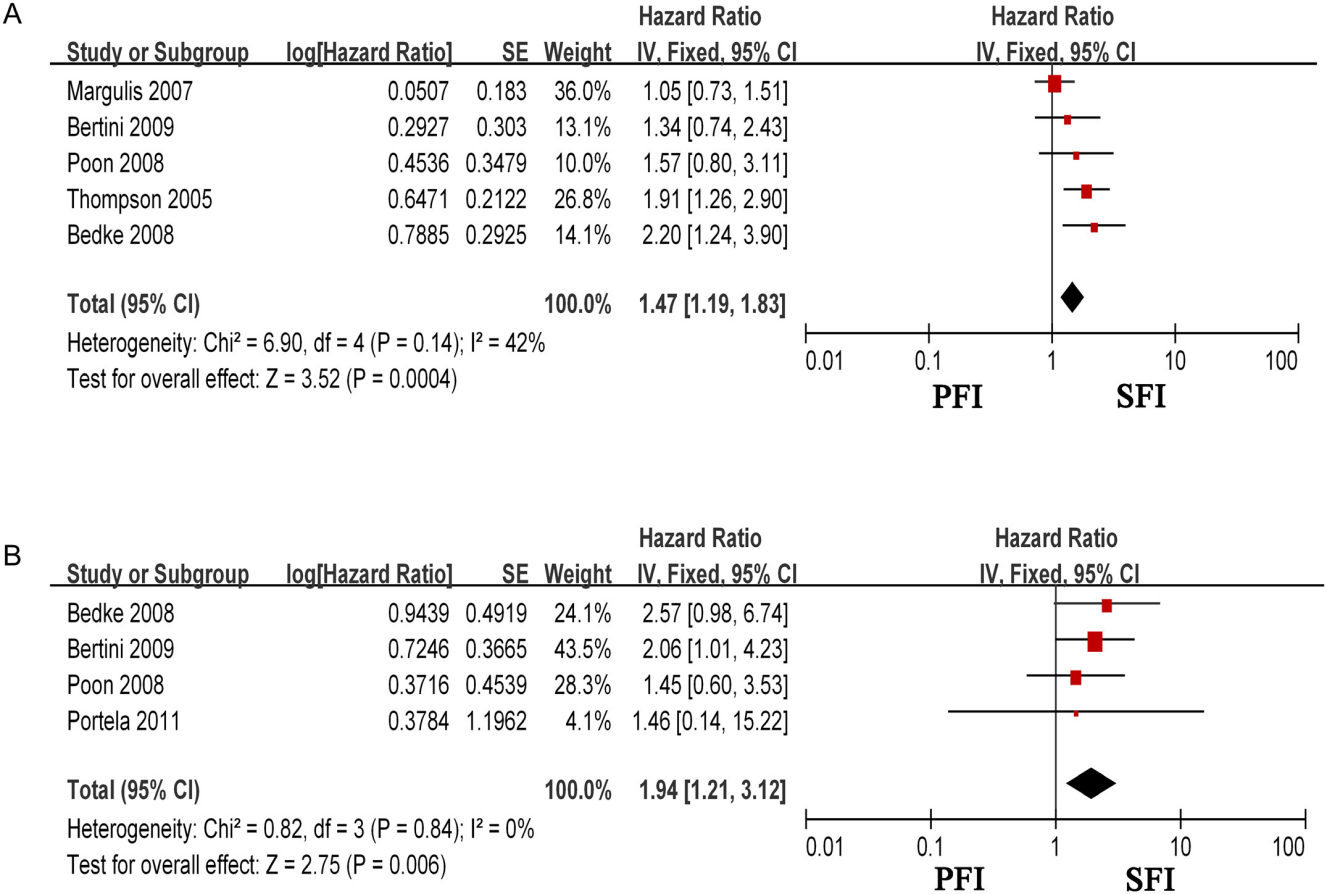
Forest plot of the hazard ratio (HR) for the association of SFI with cancer specific survival (CSS) of (A)overall T3a renal cell carcinoma patients and (B)T3aNx/N0M0 patients. HR>1 implied poor survival, and SFI was significantly associated with worse CSS. CI: confidence interval; SE: standard error.

Our study showed that SFI was significantly associated with worse CSS when compared to PFI in overall T3a patients (HR = 1.47, 95% CI = 1.19–1.83, p = 0.0004) (Fig 2A). Moderate heterogeneity was found (I^2^ = 42%, p = 0.14).

In order to analyze if our pooled estimation of the prognostic value of SFI was impacted by local and distal metastasis status, we performed a subgroup analysis focusing only on pT3aNx/N0M0. Data from four studies that reported on SFI and CSS in pT3aNx/N0M0 RCC, was pooled in the subgroup meta-analysis. As shown in Fig 2B, in the pT3aNx/N0M0 subgroup, SFI was associated with worse CSS, with a pooled HR of 1.94 (95% CI = 1.21–3.12). No heterogeneity was found (I^2^ = 0%, p = 0.84)

### Association between SFI and clinicopathologic parameters

**Fig 3:**
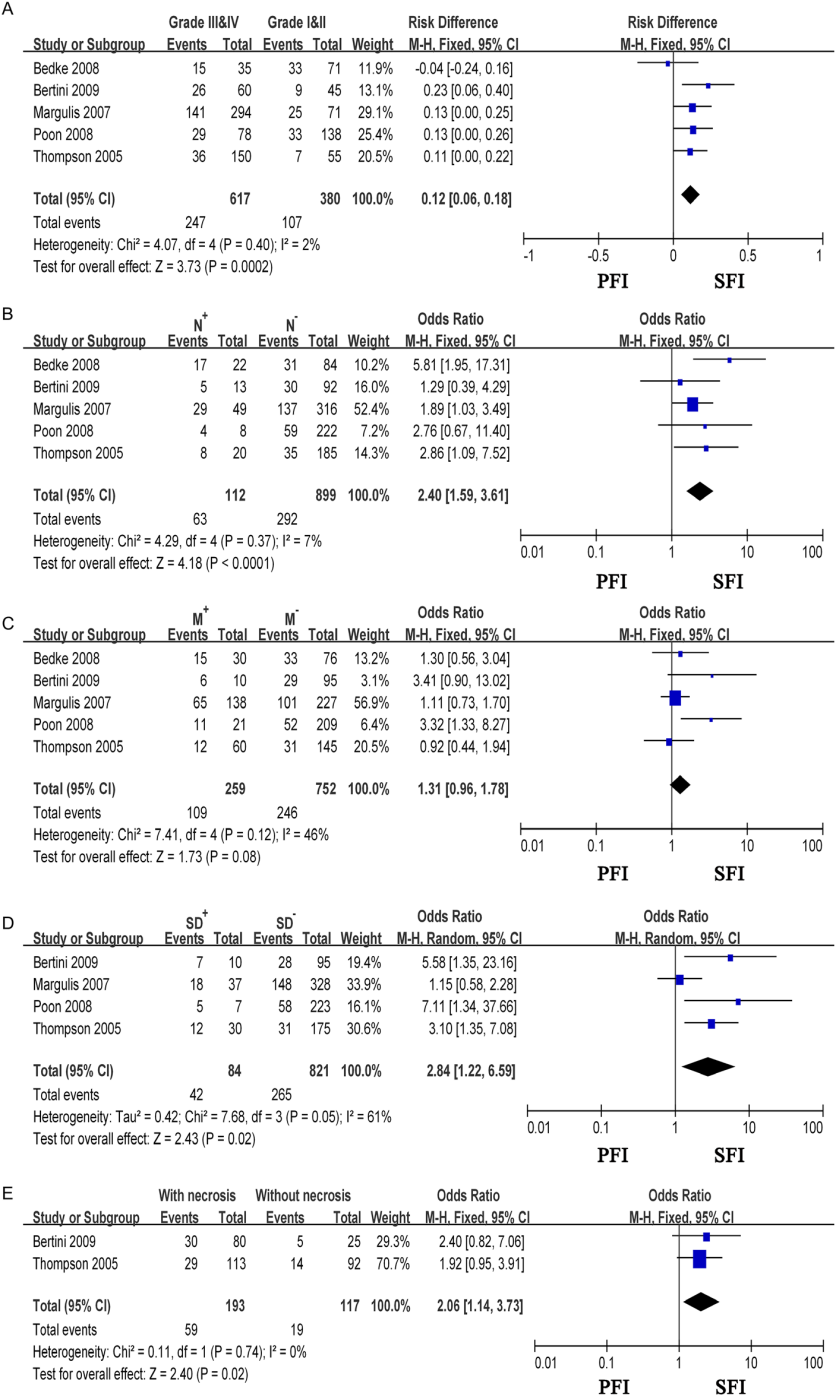
Meta-analysis of the association between SFI and clinicopathological parameters in overall T3a RCC. (A)Grade; (B) N status; (C) M status; (D) Sarcomatoid differentiation; (E) Tumour necrosis. N^+^: local lymph nodes positive; N^−^:local lymph nodes negative; M^+^: with distal metastasis; M^−^: without distal metastasis; SD^+^: with sarcomatoid differentiation; SD^−^: without sarcomatoid differentiation; CI: confidence interval; SE: standard error.

Tumors with SFI had a greater possibility of presenting as a higher grade tumor (OR = 1.76, 95% CI = 1.29–2.40, p = 0.0003), with lymph nodes metastasis (OR = 2.40, 95% CI = 1.59–3.61, p<0.0001), sarcomatoid differentiation (OR = 2.84, 95% CI = 1.22–6.59, p = 0.02) and tumour necrosis (OR = 2.06, 95% CI = 1.14–3.73, p = 0.02). Conversely, patients with PFI were associated with less aggressive clinicopathologic parameters. The results are shown in Fig 3. However, there was no significant association between SFI and distant metastasis (Fig 3C), histological type and presenting symptoms (data not show).

### Publication bias

**Fig 4:**
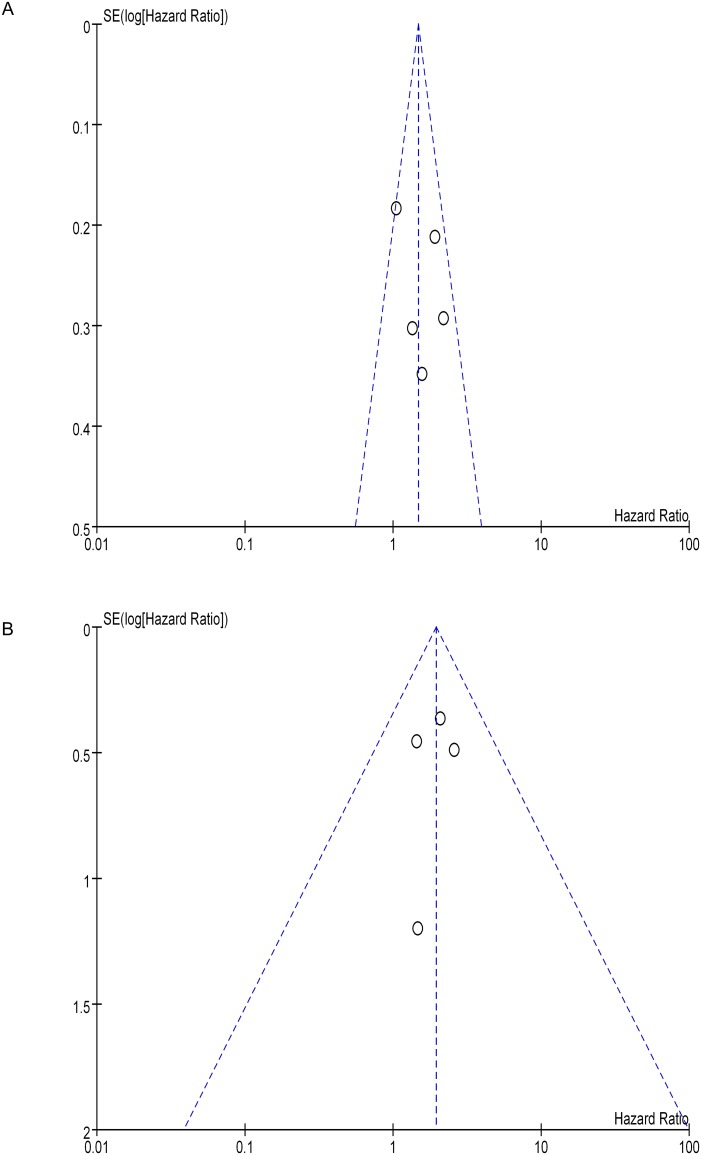
Funnel plot for all studies included in this meta-analysis. (A) Funnel plot assessing SFI and cancer specific survival (CSS) in T3a renal cell carcinoma patients. (B) Funnel plot assessing SFI and CSS in T3aNx/N0M0 patients. SE: standard error.

As shown in Fig 4, the shape of the funnel plot was symmetrical, suggesting no obvious publication bias in the included studies for CSS. Furthermore, Begg’s test and Egger’s test also showed no significant publication bias in the studies for either the whole group (Begg’s test, p = 0.806, Egger’s test, p = 0.428) or the T3aNx/N0M0 group (Begg’s test, p = 1.000, Egger’s test, p = 0.767).

### One-removed analysis

In order to gauge the stability of our results, a one-removed analysis was conducted, in which one study was omitted at a time. Table 2 shows the results of the analysis. In the 5 studies that reported overall T3a CSS, the corresponding pooled HR for CSS did not significantly change, regardless of which study was deleted, suggesting that the result was robust. However, the pooled HR appeared to be unstable in the 4 studies that reported CSS in T3aNx/N0M0 subgroup. When Bertini's [4] study was omitted, the pooled HR became insignificant.

**Table 2:** One-removed Analysis for CSS.

	Study omitted	HR (95% CI)	P Vaule
**Overall Group**	Bertini^[^^4^^]^	1.57 (1.09, 2.27)	0.02
	Poon^[^^8^^]^	1.52 (1.07, 2.17)	0.001
	Bedke^[^^6^^]^	1.38 (1.09, 1.74)	0.007
	Margulis^[^^7^^]^	1.78 (1.36, 2.33)	<0.0001
	Thompson^[^^5^^]^	1.34 (1.04, 1.72)	0.02
**T3aNx/N0M0 Group**	Bertini^[^^4^^]^	1.85(0.99, 3.48)	0.06
	Poon^[^^8^^]^	2.18(1.25, 3.81)	0.006
	Bedke^[^^6^^]^	1.78(1.03, 3.06)	0.04
	Portela^[^^14^^]^	1.96(1.21.3.19)	0.006

CSS: cancer specific survival; HR: hazard ratio; CI: confidence interval.

## Discussion

The primary purpose of staging malignancy is to better assess the behavior and prognosis of the staged tumor. In order to better accomplish this, TNM staging for RCC is amended from one edition to the next. Adrenal invasion, for example, was classified as a T3a disease in the 6^th^ edition TNM staging [1], however, increasing studies have reported a worse prognosis with adrenal invasion than with other T3a diseases. Some studies have reported an oncological outcome for adrenal invasion disease that is comparable to T4 or metastatic disease [15–17]. Hence, the 7^th^ edition, correspondingly reclassified adrenal invasion as a T4 or M1 disease [2]. though the latest 7^th^ edition TNM staging system is under controversy, especially in regards to the T3a group.

T3a RCC includes two types of fat invasion: perinephric fat invasion (PFI) and renal sinus fat invasion (SFI). Theoretically, T3a RCC patients with SFI have a higher risk of developing metastatic disease due to the presence of abundant veins and lymphatics in the sinus, which may lead to a worse prognosis [3]. However, after systematically reviewing the literature, we were unsatisfied with the unconvincing results. Several studies have found a compromised survival rate in SFI patients when compared to PFI patients [5–6]. For example, Thompson and colleagues found that patients with SFI were 63% more likely to die of RCC compared to those with PFI [5]. In contrast, some studies found that there was no significant difference in prognosis between PFI and SFI T3a RCC [4, 7–8]. For instance, Margulis and colleagues reported that neither SFI nor the location of extrarenal extension were important prognostic factors in cancer specific mortality [7]. Due to the rarity of T3a fat invasion RCC, a pooled analysis with large sample size may help better demonstrate this issue.

In the current study, we performed a meta-analysis, which synthesized the results of all related studies, in order to explore the prognostic difference between PFI and SFI in T3a RCC. We found that T3a RCC patients with SFI were 1.47 fold more likely to die of cancer, suggesting that SFI is associated with a worse prognosis compared with PFI. In order to eliminate the impact of local and distal metastasis, we performed a subgroup analysis focused only on pT3aNx/N0M0 patients. The result showed that the prognosis of SFI in this subgroup was also worse than PFI with a HR of 1.94. The above results suggest that there is worse CSS in patients with SFI pT3a RCC. The number of included studies is moderate, therefore, a future study that is ideally multi-center collaborative, with robust patient numbers will be required to validate our findings.

Along with the limited number of T3a RCC fat invasion patients in each included study, there may be other factors that contributed to the conflicting results in literature. Firstly, some fat invasion cases may have been ignored during pathological diagnosis. In the current study, we found that fat invasion diagnosis rate varies among studies, from 5.1% to 18.5%. Without careful examination, some T3a SFI cases were misdiagnosed as T1 or T2. Lack of a central pathology review may also affect the detection rate of fat invasion disease in different studies [18–20]. Secondly, some studies only focused on radical nephrectomy patients, however others included both radical and partial nephrectomy cases. Although in localized RCC, radical and partial nephrectomy acquire similar oncological outcome [21], T3a RCC may result in unpredictability. However, the included studies didn't provided enough data for a subgroup analysis of radical versus partial nephrectomy. Again, a prospective study with a central pathology review and rigorous control of surgery type may be needed to answer this controversial question in a more determinate manner.

This study is the only meta-analysis on the difference in prognosis between SFI and PFI for T3a RCC as far as we are aware. In the absence of larger well-controlled series comparing the prognostic difference between the two rare groups, our study supports that SFI is associated with worse CSS, however, certain limitations in our study must be addressed. Firstly, there was moderate heterogeneity when the HR of the overall T3a group was pooled. Subgroup analysis in T3aNx/N0M0 showed no heterogeneity between studies, which suggested that the metastasis status may contribute to the heterogeneity. Even so. it was difficult to explain this heterogeneity in our meta-analysis due to the limited number of eligible studies. Secondly, we preferentially extracted HR from multivariable analysis, which was adjusted for other factors. However, the adjusted factors were not the same in HRs that were directly extracted from multivariate Cox analysis. We used the Kaplan-Meier curves to estimate the HRs in studies that did not directly provide HRs. All of these factors, more or less, contributed to the observed heterogeneity. Thirdly, although one-removed analysis showed stability of our findings in the overall group, the HR results in the T3aNx/N0M0 subgroup were not that stable. When Bertini's [4] study was omitted, the pooled HR became insignificant. This was also due to the small study number that was analyzed. Only the Bertini's study showed a statistically significant positive association between SFI and poor CSS in this subgroup. Finally, our meta-analysis only included published studies written in the English language, therefore unpublished or non-English studies were not identified in our literature search and thus were not included in this analysis.

In summary, the present meta-analysis suggests that SFI is associated with worse CSS than PFI in patients with pT3a RCC. Given the limitations listed above, our conclusions from this study need to be interpreted with caution. Future large series studies with rigorously designed methodology are required to verify our results.

## Supporting Information

S1 PRISMA Checklist PRISMA 2009 Checklist. (DOC)
